# A Portable Measurement Device Based on Phenanthroline Complex for Iron Determination in Water

**DOI:** 10.3390/s23031058

**Published:** 2023-01-17

**Authors:** Samuel Fernandes, Mouhaydine Tlemçani, Daniele Bortoli, Manuel Feliciano, Maria Elmina Lopes

**Affiliations:** 1 Department of Mechatronics Engineering, School of Science and Technology, Universidade de Évora, 7000-671 Évora, Portugal; 2 Instrumentation and Control Laboratory (ICL), Insititute of Earth Sciences (ICT), Universidade de Évora, 7000-671 Évora, Portugal; 3 Physics Department, School of Science and Technology (ECT), Universidade de Évora, 7000-671 Évora, Portugal; 4 Earth Remote Sensing Laboratory (EaRSLab), Institute of Earth Sciences (ICT), Universidade de Évora, 7000-671 Évora, Portugal; 5 Centro de Investigação de Montanha (CIMO), Instituto Politécnico de Bragança, Campus de Santa Apolónia, 5300-253 Bragança, Portugal; 6 Laboratório Associado para a Sustentabilidade e Tecnologia em Regiões de Montanha (SusTEC), Instituto Politécnico de Bragança, Campus de Santa Apolónia, 5300-253 Bragança, Portugal; 7 Department of Chemistry and Biochemistry, School of Science and Technology (ECT), Universidade de Évora, 7000-671 Evora, Portugal

**Keywords:** iron, optical sensor, portable device, water, absorbance, opto-electronic, chemistry, environment, human health

## Abstract

In this work, a newly developed self-contained, portable, and compact iron measurement system (IMS) based on spectroscopy absorption for determination of Fe^2+^ in water is presented. One of the main goals of the IMS is to operate the device in the field as opposed to instruments commonly used exclusively in the laboratory. In addition, the system has been tuned to quantify iron concentrations in accordance with the values proposed by the regulations for human consumption. The instrument uses the phenanthroline standard method for iron determination in water samples. This device is equipped with an optical sensing system consisting of a light-emitting diode paired with a photodiode to measure absorption radiation through ferroin complex medium. To assess the sensor response, four series of Fe^2+^ standard samples were prepared with different iron concentrations in various water matrices. Furthermore, a new solid reagent prepared in-house was investigated, which is intended as a “ready-to-use” sample pre-treatment that optimizes work in the field. The IMS showed better analytical performance compared with the state-of-the-art instrument. The sensitivity of the instrument was found to be 2.5 µg Fe^2+^/L for the measurement range established by the regulations. The linear response of the photodiode was determined for concentrations between 25 and 1000 µg Fe^2+^/L, making this device suitable for assessing iron in water bodies.

## 1. Introduction

Water is one of the most important natural resources for humans in everyday life. Fresh water can be used in multiple activities, such as in agriculture, industry, domestic settings, recreation, energy production, aquaculture, and livestock watering [1]. Providing safe water for these activities, especially those involving life, is essential [2]. A concentration of pollutants above recommended quality standards (200 µg Fe^2+^/L), as defined by Directive 98/83/EC, Drinking Water Directive (DWD) [3], can pose a major risk to living beings [4,5,6,7] since water can act as a transport vector of natural or anthropogenic pollutants, toxins, and pathogenic microorganisms, such as viruses, bacteria, fungi, protozoa, and worms [8,9]. A high concentration of iron in water bodies can cause algal blooms, which can lead to increased demand for oxygen, higher production of neurotoxins, and higher fish mortality as the iron builds up in the internal organs. A recent study showed a relationship between high concentrations of iron in rivers and decreased species diversity and abundance of periphyton, benthic invertebrates, and fishes [10]. Iron is a trace chemical element that is essential to every organism [11]; it plays a major role as a constituent of enzymes and on oxygen-carrying proteins, such as hemoglobin and myoglobin. However, intake of high concentrations of iron is linked to harmful effects for people. As people age, deposition of iron in different parts of the brain can lead to abnormal cognitive function and behavior [12]. Some studies link iron accumulation in the human brain to accelerating progress of Alzheimer’s disease [13,14,15], or to neurodegenerative conditions such as Parkinson’s disease and multiple system atrophy [16,17]. Iron is also positively correlated with arteriosclerosis [18,19] and diabetes mellitus [20,21]. Excess iron in drinking water can also produce an unpleasant metallic taste [22].

The methodologies to quantify iron in water bodies involve complex logistics, requiring coordination between the laboratory and field operators, presence and use of reagents for sample preservation, and previous preparation of materials to accommodate samples during transport to the laboratory [23,24,25]. In some cases, it takes days between sample collection and sample analysis. If there is a fault during this period, the properties of the sample may be changed and it will no longer be representative of the water body from where it was collected. These laboratory methodologies also require instruments that involve high acquisition and maintenance costs, such as inductively coupled plasma (ICP) [26,27]. Flow injection using colorimetry, for example, with its low-cost maintenance, makes the process less expensive, but the initial investment is significantly high, it has a large form factor, and it cannot be easily carried into the field [28,29]. Some commercial solutions capable of performing in situ measurements have emerged in recent years in devices on the market [30], but they are for single parameter determination as they only allow determination of one chemical analyte per instrument even when the wavelength reported between instruments for different parameters is the same. Since these are proprietary commercial devices, they are hard-wired for only one preset wavelength, and it is not possible for the user to calibrate them for determination of other substances. These also cost more than USD 950 per unit, which inhibits widespread adoption in large-scale monitoring programs, and, as a result, the scientific community keeps looking at economical and more affordable alternatives.

The recent development and mass production of embedded systems [31,32] and optoelectronic devices, such as diodes and photodiodes, have enabled implementation of affordable and open-source devices. The first time the term paired emitter detector diode (PEDD) was used dates back to 1993 [33]. Since then, this methodology of paring a diode and photodiode has been successfully used for determining water quality parameters (nitrate, nitrogen [34,35], turbidity [36], phosphate [37], phosphorous [38], paraquat and diquat [39], fluoxetine and citalopram [40], and chromium [41]). Use of novel signal processing algorithms that can replace some hardware leads to better instrument performance at a lower cost. These recent developments have allowed for customization of solutions for particular problems or applications. In some cases, the new systems can replace commercial instruments with the same or higher performance [42]. In the recent literature, a similar approach to Fe^3+^ determination to the one developed in this work appears [43] that uses the thiocyanate method composed of open-source hardware and software. However, it produces weak color intensity, fading in a short period of time, typically 15 to 30 min [44]. The instrument also uses a built-in white-light-emitting diode (LED) as the photon source. This implies that the photons are reflected to the sensor using the material on the 3D-printed cuvette support, which can cause high variation in the scattering geometry [45,46] and interference of other substances in determination of iron.

In this work, we present the development, setup, characterization, and calibration of a colorimetric instrument, the iron measurement system (IMS), for determination of Fe^2+^ in water samples based on the phenanthroline standard method [47]. This method uses hydroxylamine, acetic acid, sodium acetate, and phenanthroline to react with the Fe^2+^ present in the water sample to produce orange coloration.

The main goals of the IMS are:

- Operate the device in the field in standalone operation, as opposed to instruments commonly used exclusively in the laboratory, at lower cost.
- Quantify iron concentrations in accordance with the value proposed by the regulations for human consumption.
- Use of a paired diode–photodiode with three radiation channels (red, green, and blue) allows the device to determine multiple water parameters without the necessity to modify the hardware configuration.

In the following sections, the IMS is presented and fully described, highlighting the main mechanical, optical, and electronic components (Section 2.1, Section 2.2, Section 2.3 and Section 2.4). Section 2.5 describes preparation of the four series of samples utilized for sensitivity studies. Section 4 describes the IMS calibration. Section 2.5 also explains and clarifies the preparation of the homemade reagent used for on-field measurements. In Section 2.6, the theoretical aspects of the extinction law, which is the basis of the retrieval method, are presented together with the definitions of the detection and quantitation limits, which are calculated and discussed in the third section to ensure truthfulness of the retrieved concentrations. In the fourth section, all measurements performed with the IMS and the reference Nicolet Evolution 300 spectrophotometer (Thermo) on the four series of samples are presented. A comparative study on the obtained datasets is presented and discussed in detail based on an analysis of the statistical parameters obtained from comparison of corresponding absorbance measurements and determined concentrations. Section 5 includes a summary of this work, with conclusions, remarks, and ongoing and future research plans.

## 2. Materials and Methods

### 2.1. Instrument Description

**Figure 1:**
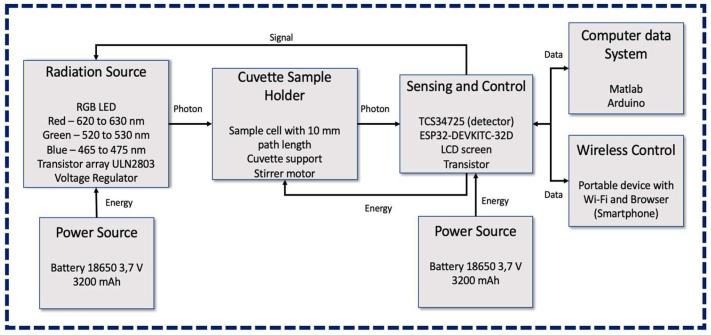
Block diagram presenting main components of IMS and representation of flow of light radiation, energy, data, and signal control through various domains in the instrument.

A block diagram showing the main components of the IMS device developed for colorimetric determination of Fe^2+^ in fresh water is presented in Figure 1. The flow of the radiation, data, energy, and control signal are presented as well. The radiation source module is equipped with a general-purpose red, green, and blue (RGB) LED, a Darlington transistor IC (UNL2803) for selection of the LED emitting channel, and a voltage regulator to maintain constant voltage coming from the energy source. The power source module for this block consists of 2 batteries of 3.7 V with 3200 mAh each. The cuvette sample holder can accommodate a cuvette of 10 mm path length containing the water samples being measured, and a stirrer motor was introduced to investigate its efficiency in shaking solvents and reagents.

**Figure 2:**
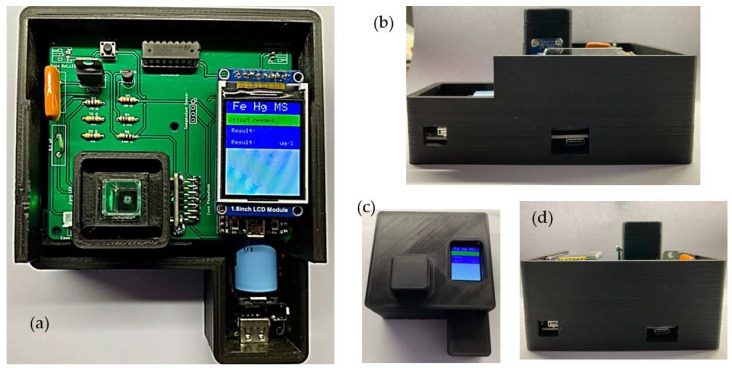
(**a**) Top view of final version of developed IMS without top cover; (**b**) right view; (**c**) top view of IMS fully assembled, with top cover and cap cuvette cover; (**d**) back view of system.

Aiming to reduce voltage fluctuations and maintain better stability of the emitted radiation, a second power source module, independent and with the same features as the first one, supplies the sensing and control bloc, which accommodates the RGBC TCS34725 sensor. The sensor has 4 channels, RGB and clear, with a dynamic range of 3,800,000:1 and an IR filter that almost blocks the detection after 700 nm; i.e., the blue, green, and red channels have maximum sensitivity at 475, 550, and 620 nm with FWHM of 104, 79, and 55 nm, respectively. The clear channel ranges from 300 to 700 nm. The RGBC sensor has 4 integrated ADCs that allow the amplified photodiode current to be converted into digital 16-bit values. The sensor works with voltage of 3.3 V and current of 235 µA. The signal gain can be set at 1X, 4X, 16X, and 60X. Integration time ranges from 2.4 to 614 ms. The sensing and control module also includes the ESP32-DEVKITC-32D microcontroller, which drives the RGB LED and the stirrer motor, allowing for temporary storage of the data coming from the TCS34725 sensor, and an LCD screen to show the measurement results to the user. The ESP32 and screen are assembled in a double stack configuration, allowing us to decrease the size of the instrument. The computer data system is simply a personal computer (PC) linked to the IMS through a USB connection, and the PC is equipped with Arduino software for programming the ESP32 board and developed MATLAB scripts for storing and processing the data. The wireless control block can be any device with standard Wi-Fi IEEE 802.11 communication and a browser to interact with a web page stored in the microcontroller. The portable device allows access to a built-in web page to set the blank water sample in the beginning of the measurement operation, then record the iron concentration and activate the stirring motor. The final version of the IMS without the upper cover for demonstration purposes is presented in Figure 2a. The right-side view of the instrument (Figure 2b) shows the two engraved entrances to charge the first power supply and the button to turn the sensing and control modules on and off. Figure 2c shows the upper view of the instrument with the top cover and the cuvette cap. Finally, Figure 2d shows the back side view of the instrument with two engraved holes to charge the second power supply and a switch to turn the RGB diode on and off. The device also includes a button to set the measurement of the blank sample and perform measurement of water samples containing Fe^2+^.

### 2.2. Sensing System Design

**Figure 3:**
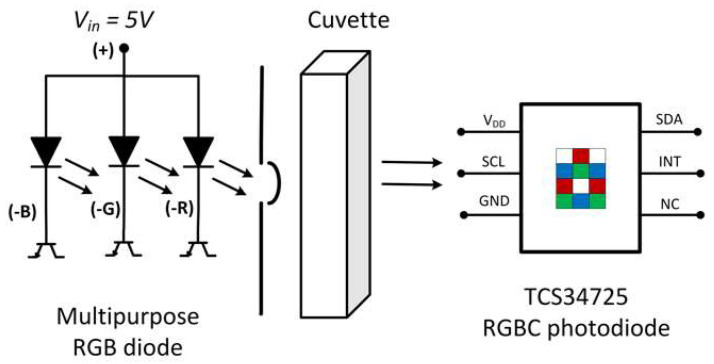
Diagram of common anode photon-emission controlled by transistor matrix and cuvette, and representation of RGBC TCS34725 photodiode.

The iron measurement system is designed to quantify the complex ferroin between Fe^2+^ and phenanthroline based on the colorimetric method. The diode and photodiode are aligned parallel on opposite sides of the cuvette support (Figure 3). The support, besides allowing these two components to be mounted, also provides insulation from the radiation of the outside environment, preventing it from interfering with the measurements.

**Figure 4:**
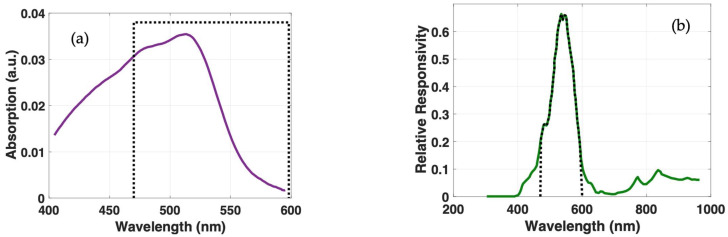
(**a**) Absorption spectrum of ferroin with maximum absorption wavelength at 514 nm and integration area by superposition of spectral range 470–600 nm (dashed black line); (**b**) overlap of wavelength ranges of photodiode (green line) with radiation emitted from green channel of RGB LED (dashed black line) with highest spectral response at 525 nm.

The absorbance spectrum of the ferroin complex formed between the Fe^2+^ and the phenanthroline in a water solution, measured using the Nicolet Evolution 300 spectrophotometer within 410 to 590 nm wavelength, is shown in Figure 4a. The maximum peak of absorbance was found at 514 nm, with a value of 0.036 A for a concentration of 200 µg Fe^2+^/L. Pairing the multi-function RGB LED with the current sensor (Figure 4b) enables determination of the ferroin by integrating the obtained signals in the 470–600 nm range, where the ferroin exhibits the highest absorbance. This ensures that interfering agents from other spectral regions will not affect the measurements.

### 2.3. Iron Measurement System Design and Setup

**Figure 5:**
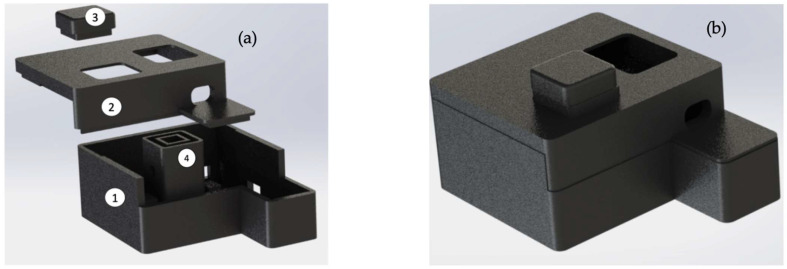
(**a**) Explosion view of 3D enclosure implemented to receive electronic components; (**b**) rendering of device closed for demonstration purposes.

The 3D-printed case was designed to accommodate all the components of the developed IMS device. It was developed and printed in 3 parts, as shown in Figure 5a, where the main components are labelled: the main body, where the electronic components are placed; the upper cover of the instrument; the cap to cover the cuvette; and the cuvette support, which can accommodate cells of 10 mm width. All parts are printed in black polylactic acid to decrease absorbance interference from the outside environment and scattering radiation indoors. The engraved hole on the structure allows excess water to be flushed out. A rendering of the instrument in a closed assembled configuration is shown in Figure 5b.

**Figure 6:**
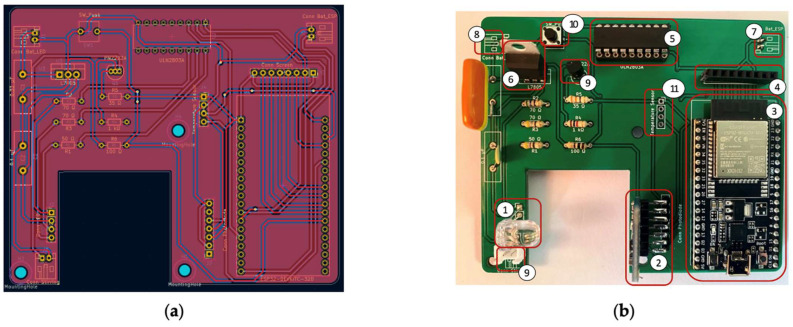
(**a**) Schematic view of developed PCB; (**b**) front view of physical PCB with main electronic components assembled.

A PCB was developed and built to accommodate all the electronic components of the IMS. Implementation of the PCB also allows the system to be developed in small form factor to reduce noise, improve the signal-to-noise ratio, and increase the portability so the IMS could be used on field campaigns without damaging the connections between components. The PCB was produced in FR 4 TG 130–140 material with a thickness of 1.6 mm and a green solder mask (Figure 6a). The final version with the main components assembled is presented in Figure 6b.

The PCB combines (1) the RGB LED, (2) the TCS34725 sensor, (3) the embedded ESP32 system, (4) an LCD connector, (5) a Darlington transistor IC to enable use of the channel of interest on the diode, (6) a voltage regulator to ensure steady constant voltage for the photon source, (7) an energy supply connector for the signal and control module, (8) an energy supply connector for the RGB diode, (9) a transistor to control the stirring motor, (10) a push button for offset and measurement start and control, and (11) a pad for optional temperature sensor installation.

### 2.4. Photon Emission Characterization

**Figure 7:**
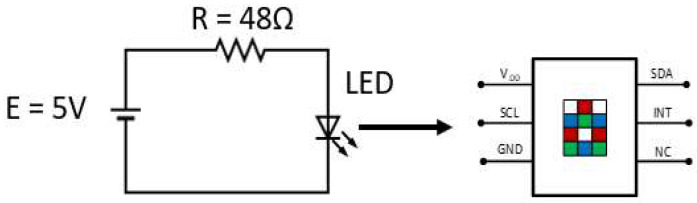
Instrument configuration used for characterization of source radiation. Left side is a schematic diagram of the RGB LED green channel; right side is a representation of the RGBC TCS34725 photodiode.

For better characterization of the photon source, sensitivity analysis based on a statistical approach was carried out. The integrated intensity of the radiation emission between wavelengths of 470 and 600 nm coming from the green channel of the RGB LED was measured as illustrated in Figure 7. The LED was supplied at 5 V with load resistance of 48 Ω for the green channel, and a TCS34725 photodiode was used to quantify the radiation reaching the sensor.

Since the maximum absorption of the ferroin complex is in the green spectral region (514 nm, as pointed out in Section 2.2), in this paper, only the green channel of the RGB radiation source is characterized. The radiation emitted from the LED green channel was acquired 5000 times to determine the stability of the signal and the performance of the TCS34725 sensor. Chi−square statistical method provides assurance of a normal distribution of radiation coming from the RGB LED green channel combined with the photodiode.

**Figure 8:**
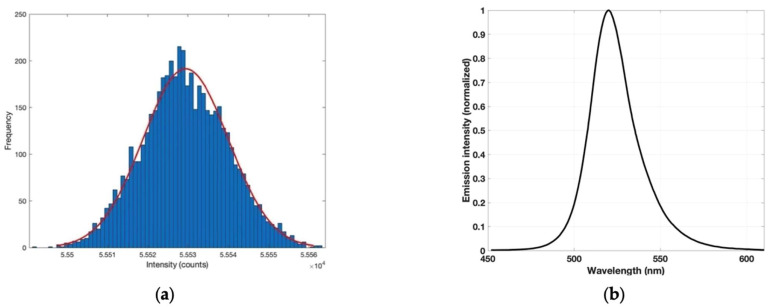
(**a**) Normal error curve distribution for green channel of diode, 5000 measurements (blue bars), and best fit of distribution (red line); (**b**) spectral energy distribution of RGB LED green channel with a peak at 520 nm.

The frequency distribution of the emitted radiation of the LED measured using the RGBC TCS34725 sensor allowed us to verify that the photometric characteristics of the two opto−electronic components followed a normal distribution (Figure 8a, blue bars). As expected from a normal distribution, the data present a typical Gaussian shape (Figure 8a, red line). The average radiation intensity recorded was 55,529 counts, with a standard deviation of 11 counts. The LED spectrum was characterized using the SNT˗BLK-CXR UV-vis spectrophotometer. The normalized emission intensity for the green channel of the LED in the spectral range 450–610 nm is presented in Figure 8b. As observed, this channel had a maximum intensity peak at 520 nm with an FWHM of 26.9 nm.

### 2.5. Chemical Reagents and Fe^2+^ Samples

All solvents and reagents were of analytical grade and prepared with distilled water. The adopted procedure is described in [47]. The following solutions were prepared:

(1) Stock solution of Fe^2+^ of 25 mg Fe^2+^/L obtained by diluting 0.0176 g of Fe(NH_4_)_2_(SO_4_)_2_•6H_2_O (Panreac) and one drop of concentrated hydrochloric acid to 100 mL.
(2) Solution of hydroxylamine (0.1 M) prepared by dissolving 10 g of NH_2_OH•HCl (Panreac) to 100 mL.
(3) Acetate buffer solution with pH 4.5, obtained by mixing 65 mL of acetic acid 0.1 M with 35 mL of sodium acetate (Panreac) 0.1 M.
(4) Phenanthroline solution (1,10−phenantroline 99+%; Acros Organics) prepared by diluting 100 mg of a homemade solid reagent (whose composition is described in the next paragraphs) and two drops of concentrated hydrochloric acid to 100 mL.

**Figure 9:**
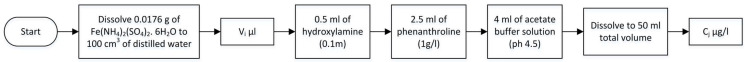
Flowchart for Fe^2+^ standard solution preparation using phenanthroline. Vi denotes volume of iron solution necessary to prepare standard sample with concentration C_j_, in µg Fe^2+^/L.

The first and second series of standard samples, in concentrations of 50, 100, 200, 500, and 1000 µg Fe^2+^/L and 25, 50, 75, 100, 125, 150, and 200 µg Fe^2+^/L, respectively, were achieved by pouring out an adequate volume of stock solution of Fe^2+^, 0.5 mL of hydroxylamine solution, 2.5 mL of phenanthroline solution, and 4 mL of acetate buffer and finishing to a total volume of 50 mL. A flowchart of the procedure used to prepare the standard samples of Fe^2+^ is presented in Figure 9.

For the third series of standard samples (25, 50, 75, 100, 125, 150, and 200 µg Fe^2+^/L), we used the previous methodology and additionally substituted distilled water with fresh water.

The analytical procedure to prepare the fourth series of standard solutions (25, 50, 100, 150, and 200 µg F^e2+^/L) involved preparing a new mix of solid reagents. The new solid mixture was prepared in 0.2 g doses for field application to 10 mL water samples. In order to keep the iron dissolved, in the proper oxidation state (+2), and totally complexed with phenanthroline, the procedure demands the presence of a buffer at an acid pH, a reducing agent, and the complexing agent. Our mixture included citric acid (PRONALAB) and calcium hydroxide (Fluka) in adequate proportions as the buffer, ascorbic acid (Merck) as the reducing agent, and phenanthroline (1,10−phenantroline 99+%; Acros Organics) as the complexing agent. The four substances were homogenized in a mortar and pestle. A mass of 0.2 g of this reagent was applied to 10 mL of iron standard solution.

### 2.6. Calibration of the IMS Instrument

The implemented IMS instrument was calibrated using the standard samples described in Section 2.5. This methodology allowed us to determine the measurement performance of the instrument for: (i) different concentrations of Fe^2+^, (ii) different water matrices (distilled/fresh water), and (iii) the proposed solid reagent. The numerical relation between the measured absorbance and standard sample concentrations was also obtained. The absorbance of the four series of water samples was measured using the typical laboratory colorimetric analysis. A blank sample with corresponding radiation intensity (*I*_0_) devoid of iron was introduced into the cuvette; then, the signal acquired by the photodiode was sent to the digital 16−bit AD converter and the digital signal to the microcontroller. An identical procedure was carried out for each standard sample *k*. Corresponding to radiation intensity (*I_k_*), the absorbance (*A_k_*) is calculated using the following expression:

(1) $$A_{k}=-\log\left(\frac{I_{k}}{I_{0}}\right)=\log\left(\frac{C_{BS}}{C_{IS}}\right)$$

where *C_BS_* is the digital counts obtained by measuring the radiation attenuation of the blank sample and *C_IS_* the value of the signal for the *k* standard sample containing known iron concentration.

The former equation is based on the Lambert–Beer extinction law, which can be written as:

(2) $$I_{k}\left(\lambda \right)=I_{0}\left(\lambda \right)\exp\left(-\sigma \left(\lambda \right)cL\right)\Rightarrow A=-\log\left(\frac{I_{k}\left(\lambda \right)}{I_{0}\left(\lambda \right)}\right)k=1,2,\ldots ,n$$

where *σ* is the molar absorptivity of the analyte, *λ* is the wavelength of interest, *c* is the molar concentration of the analyte, *L* is the phat length of the cuvette, and *n* is the number of samples. Therefore, reasonably assuming that the integrated values provided by the IMS in digital counts are directly proportional to the spectral values, Equation (1) is consistent.

The function required to determine the amount of Fe^2+^ from the absorbance readings was obtained as the linear best fit of the experimental data to a linear function, as shown in the following expression:

(3) Y=βX+ε

where *Y* denotes the concentration of Fe^2+^ in the water samples, *X* is the Fe^2+^ absorbance measured by the photodiode, and *β* and *ε* are the parameters of the linear model.

The limit of detection (LOD) and the limit of quantitation (LOQ) were determined based on the calibration curve using Equations (4) and (5), respectively, following the guidelines proposed by the International Committee on Harmonization [48]:

(4) LOD=3.3σ/S

where *S* denotes the standard error from the regression analysis (where *n* = 30) and *σ* is the slope coefficient.

(5) LOQ=10σ/S

The measurement performance of the IMS instrument was evaluated against an optical reference instrument on 30 replicants per water solution. This approach was implemented using the Nicolet Evolution 300 UV-vis spectrophotometer (Thermo Fisher Scientific, Waltham, MA, USA) controlled by the provided VisionPro PC control software. The main characteristics of the spectrophotometer are presented in Table 1. A multiple statistical methodology approach was applied to establish the IMS instrument performance, including the best fit of the linear regression, R-square coefficient, and Pearson’s correlation.

## 3. Sensitivity Analysis

A sensitivity analysis was carried out to compare the accuracy and precision of the developed instrument against the laboratory bench top spectrophotometer and to determine its sensitivity. The measurement performance of the IMS instrument was evaluated against the reference Nicolet Evolution 300 optical spectrophotometer according to combined standard uncertainty methodology proposed by the Joint Committee for Guides in Metrology [48]. The uncertainty of the standard solutions, the retrieved values of Fe^2+^ concentration, and the measurements of precision (Prec.) and uncertainty (Uncert.) for the IMS and Nicolet Evolution 300 in the first and second series of measurements are shown in Table 2. The relative error of 1% in the standard sample concentrations is related to their preparation. For the first and second series of measurements, the mean IMS uncertainty was ±5 and ±5.6 µg Fe^2+^/L, respectively, while, for the Nicolet Evolution 300, the uncertainty was ±16.6 and ±28.2 µg Fe^2+^/L, respectively. The IMS had a sensitivity of 7.06 and 2.57 µg Fe^2+^/L in the first and second series, respectively. Overall, for these two series of measurements, the IMS outperformed the Nicolet Evolution 300. The accuracy of the Nicolet Evolution 300 is not well suited for low concentrations as the uncertainty was higher than the measured value. The IMS presented a maximum absorbance overestimation of 16.6% compared to the Nicolet Evolution 300. The *t*−test comparing the slopes of the experimental values showed *p*−values of 0.92 for the first series and 0.96 for the second series of measurements, considering α = 0.05, indicating a linear relation between the two instruments regarding measurement of iron standard solutions.

The uncertainty analysis for the third and fourth series of measurements is presented in Table 3. The IMS presented mean uncertainty values of ±2.0 and ±9.0 µg Fe^2+^/L, respectively, which are smaller than the values for the Nicolet Evolution 300, ±21.7 and ±27.4 µg Fe^2+^/L, respectively. Regarding the sensitivity of the IMS, in the third series, the value was 3.96 µg Fe^2+^/L, and, in the fourth series, it was 2.48 µg Fe^2+^/L. Both instruments exhibited increased uncertainty in the results using the developed solid reagent (fourth series). This suggests the need to improve this reagent. However, the results can be considered satisfactory at this first stage. As observed in the previous results, the new device was able to outperform the reference spectrophotometer. The *t*-test comparing the slopes of experimental values showed *p*-values of 0.89 for the first series and 0.86 for the second series of measurements considering α = 0.05, demonstrating a highly linear relation between the two devices for the iron solution standard measurements.

Sensitivity analysis of both instruments allowed us to compare their performance under the same conditions. The IMS was able to reproduce the results of the benchtop spectrophotometer and in general presented better precision and accuracy. Overall, in the four series of measurements, the IMS achieved higher precision and accuracy than the Nicolet Evolution 300. In the second and third series, at a concentration of 25 µg Fe^2+^/L, the instrument presented lower precision and accuracy, evidencing its detection limit. This behavior was not observed in the fourth series, which may be related to the new solid reagent. The *t*-test comparing the results of the two instruments showed that they have the same slope, indicating good linear agreement between the two instruments for the measurements of the standard iron solution. Regarding sensitivity, it was observed that, by reducing the range of measurement from the first series to the second, the sensitivity of the instrument increased by a factor of almost 3, which can be considered significant for determination of iron in the range of concentrations established by the regulations.

## 4. Results and Discussion

**Figure 10:**
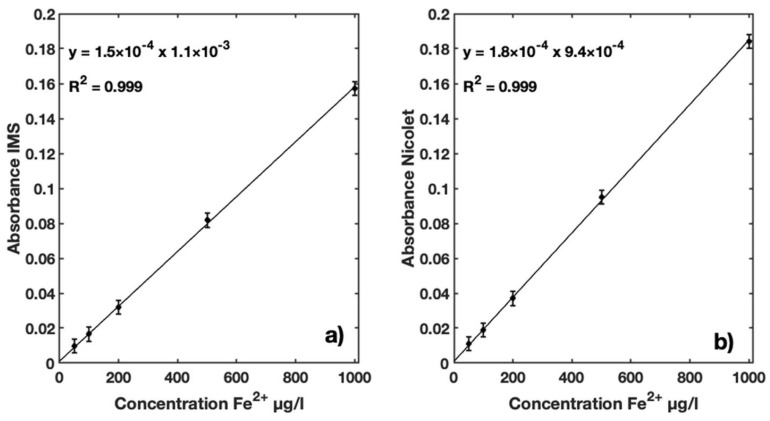
(**a**) Calibration function for Fe^2+^ ranging from 50 to 1000 µg/L using IMS instrument, with integration wavelengths between 470 and 600 nm; (**b**) calibration function for Fe^2+^ ranging from 50 to 1000 µg/L using Nicolet Evolution 300 spectrophotometer absorption coefficients at 525 nm.

The first series of measurements performed with the IMS used standard solutions in concentrations ranging from 50 to 1000 µg Fe^2+^/L. The absorbance values obtained for the iron concentrations were in the range 470–600 nm for the IMS (Figure 10a) and 525 nm for the Nicolet Evolution 300 spectrophotometer (Figure 10b).

For both instruments, the absorbance was found to vary linearly with increasing iron concentration in the standard solutions. Both instruments presented R^2^ = 0.999, indicating a good correlation between Fe^2+^ concentration and measured absorbance. The slope of linear regression for the IMS is 1.5 × 10^−4^ with an intercept of 1.1 × 10^−3^, while the slope for the Nicolet Evolution 300 is 1.8 × 10^−4^ with an intercept of 9.4 × 10^−4^.

**Figure 11:**
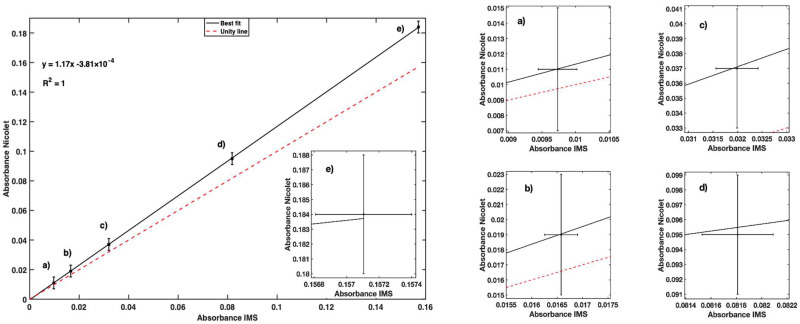
Fe^2+^ absorbance for different standard samples obtained by IMS instrument and reference Nicolet Evolution 300 spectrophotometer. Zoom-in of points is provided for better representation of error bar. For each point on the left chart, a corresponding magnified chart is presented on the right.

The agreement in measurements between the two instruments can be seen in the graph of Figure 11. A Pearson coefficient correlation of 1 with a *p*-value of 1.37 × 10^−10^ was found for the linear relation between the IMS and the reference instrument, with a coefficient of determination R^2^ = 1. Magnified views of the absorbances of 50, 100, 200, 500, and 1000 µg Fe^2+^/L are presented in order to compare the uncertainty of both measurements (Figure 11). The results allow us to conclude that the uncertainty of the IMS is significantly smaller than that of the spectrophotometer.

The unity line in the plot on the left in Figure 11 highlights the overestimation of 14.6% compared with the absorbance values measured with the Nicolet Evolution 300.

The mean absorbance values for both instruments and the calculated variance for the IMS for each standard sample of the first series of measurements are displayed in Table 4. The numerical results show very similar average absorption within the same order of magnitude for the two tested devices. For the reference Nicolet Evolution 300 spectrophotometer, the global standard uncertainty in absorbance is 0.004 A according to the instrument datasheet.

**Figure 12:**
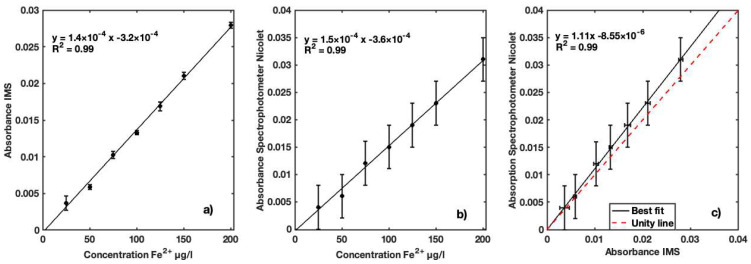
(**a**) Calibration curve for absorption coefficients of Fe^2+^ concentrations in distilled water ranging between 25 and 200 ug Fe^2+^/L using IMS; (**b**) calibration curve for absorption coefficients of Fe^2+^ concentrations in distilled water for same concentration range presented using Nicolet Evolution 300 spectrophotometer; (**c**) best fit of absorption coefficients measured by both devices to determine their performance.

With the second series of measurements, we aimed to study the performance of the IMS in a range of concentrations lower than 200 µg Fe^2+^/L. This enabled better assessment of the sensor response within a concentration range below the quality recommended guidelines. The IMS absorbance values for the concentration of Fe^2+^ in seven iron standard samples ranging from 25 to 200 µg Fe^2+^/L present a linear response, as presented in Figure 12a. A Pearson correlation coefficient of 0.99 for a *p*-value of 5.16 × 10^−10^ was determined for the linear relation between the two instruments. As expected, the absorbance measured with the reference Nicolet Evolution 300 spectrophotometer showed a linear response for all iron standards (Figure 12b). A Pearson correlation coefficient of 0.99 was determined for the linear relation between the two instruments. Good linearity with R^2^ = 0.99 between the two instruments (Figure 12c) was achieved in the range of 25 to 200 µg Fe^2+^/L. Maximum overestimation of IMS absorbance values of 9.6% relative to the Nicolet Evolution 300 was found.

In the third series of measurements, distilled water was replaced with water collected from a dam. Natural water samples were spiked with different concentrations of Fe^2+^, with the aim of determining the performance of the instrument in measuring real samples in water bodies. This procedure is performed to verify whether fresh water would interfere with measurement. The average absorbance of 30 measurements recorded with the IMS in contrast to the absorbance measured by the Nicolet Evolution 300 at a wavelength of 525 nm is shown in Table 5; the variance of the IMS is also given. Increasing absorbance of the two instruments is observed with increasing concentration of Fe^2+^ in the water solution. The results show very similar values for both instruments for the same standard sample. Iron recovery was calculated by comparing the results obtained with iron concentrations added to the natural water solution. According to the standard methods for examining water and wastewater, recovery values were found to range between 98 and 110%, which is considered acceptable recovery in terms of validation.

**Figure 13:**
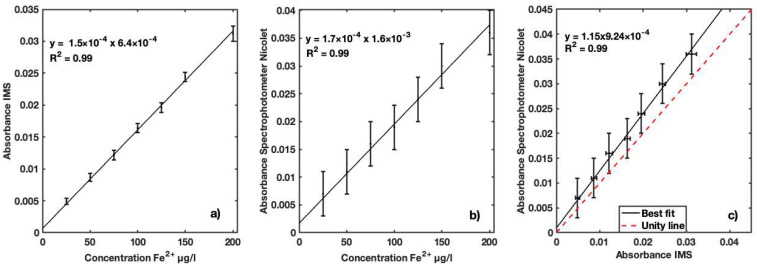
(**a**) Calibration curve of Fe^2+^ for IMS instrument for natural freshwater samples collected in a dam in concentrations ranging between 25 and 200 µg/L; (**b**) calibration curve of Fe^2+^ for same sample concentrations for reference Nicolet Evolution 300 spectrophotometer; (**c**) best fit of absorption coefficients measured with the two instruments for performance comparison.

Absorbance measurements of samples with added Fe^2+^ ranging between 25 and 200 µg/L by the IMS instrument demonstrated good linearity with the standard concentrations, presenting R^2^ = 0.99 (Figure 13a). Determination with the reference spectrophotometer using the phenanthroline method for natural water (Figure 13b) showed a good relation (R^2^ = 0.99) between the absorbance and Fe^2+^ concentrations at a wavelength of 525 nm. A Pearson coefficient correlation of 0.99 for a *p*-value of 2.99 × 10^−8^ for the linear relation between the two instruments was obtained (Figure 13c). As previously noted, in these measurements, the uncertainty was significantly smaller for the IMS than the reference Nicolet Evolution 300 spectrophotometer.

The results for the IMS, averaged over 30 measurements per sample, the measurement absorbance for the Nicolet Evolution 300, and the variance for the IMS are presented in Table 6. Absorbance values vary linearly with increasing Fe^2+^ concentration in the prepared standard samples. A slight increase in absorbance values is noted compared with the results obtained in the second series, indicating the presence of Fe^2+^ in the original freshwater samples from a dam.

**Figure 14:**
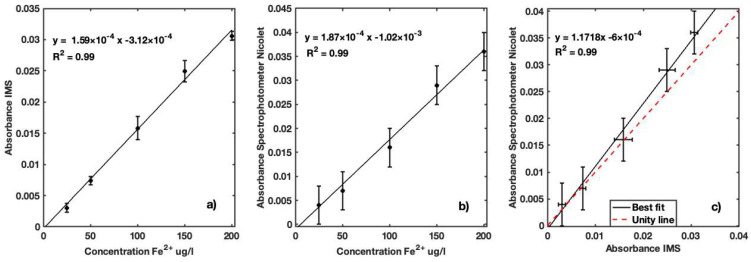
(**a**) Calibration curve of Fe^2+^ for developed solid reagent using IMS instrument for concentrations ranging between 25 and 200 µg Fe^2+^/L in distilled water; (**b**) calibration curve of Fe^2+^ for reference Nicolet Evolution 300 spectrophotometer for same concentration range; (**c**) best fit of absorbances measured with the two instruments for performance comparison.

The fourth series of measurements demonstrated the feasibility of the solid reagent for determination of iron in water samples. A high linear correlation (Figure 14a) was obtained between the absorbance and standard solutions of Fe^2+^, indicating good performance of the IMS in determining iron using the new solid reagent. The same performance was obtained with the reference spectrophotometer, highlighting the effectiveness of the reagent (Figure 14b). The comparison of absorbance measured by the two instruments showed very good linearity and performance (Figure 14c). A Pearson coefficient correlation of 0.99 with a *p*-value of 1.71E-5 was obtained for this comparison. This relation indicates good performance of the developed reagent and the instrument. In the absorbance measured with the IMS, a maximum overestimation of 13.8% relative to the Nicolet Evolution 300 was observed. The LOD and LOQ values were 8.07 and 24.45 µg Fe^2+^/L, respectively, which indicates the instrument’s ability to determine and assess iron concentration levels according to regulatory standards.

The mean absorbance over 30 measurements obtained with the IMS and one sample measurement with the reference spectrophotometer for each standard sample prepared with the solid reagent are presented in Table 7. The variance per sample with the IMS is also shown. The obtained absorbance for both instruments was very similar in value and magnitude. This shows the feasibility of using the IMS and the solid reagent to determine the concentration of Fe^2+^ in water samples.

A good degree of correlation between the experimental data obtained for the four series of measurements is highlighted. The first series covered a broad range of concentrations between 50 and 1000 µg Fe^2+^/L, with a high correlation. The concentration of Fe^2^ in the water solutions was confirmed by the benchtop spectrophotometer, and the relation between absorbance measured by the two instruments was considered very high. The second series of measurements, in the range of concentration between 25 and 200 µg Fe^2+^/L, not only showed a high correlation between the absorbance and the standard solution but also enabled a considerable increase in instrument performance. The third series of measurements, using natural water, made it possible to test the instrument on real samples spiked with iron standards. The recovery percentage (%Recovery) of the designed instrument, shown in Table 5, is considered acceptable according to standard methods. The final series of measurements enabled us to study the ready-to-use solid reagent prepared in-house. The results of the first tests are promising as a high correlation between the standard iron solutions and the absorbance was found for the two instruments. In the future, a more dedicated study of this new reagent using real water samples will be developed. Our method has the potential to be a valuable tool for iron detection in water samples due to its high accuracy, precision, sensitivity, affordability, usability, and visualization of the measured parameter.

A summary of the different instruments found in the market and scientific publications to determine different water quality parameters is presented in Table 8.

These instruments follow the pairing diode–photodiode methodology. The cost of the commercial instruments was to be found above and below compared to the IMS; however, some do not offer the same multiplicity of analyzed parameters, wireless communication, and remote control. The instruments found in the scientific bibliography can present a lower estimated price, but, as with the Smart Trubidimeter [49], they do not report all the costs associated with all the components of the system; only prices of principal components are presented. Moreover, these devices do not present wireless and remote-control communication.

**Table 8:** Summary of the different commercial solutions and scientific publications dedicated to determination of water quality parameters.

Instrument	Cost	Radiation Source	Measured Parameter	Sensitivity	Measuring Range	Precision	Accuracy	Recalibration	Power Source	Reagent	Wireless Communication	Remote Control
IMS	EUR 75	LED	Iron (possibility to extend to more parameters)	2.57–7.06 mg Fe^2+^/L	0–1 mg Fe^2+^/L	±1.0 – ±13.0 μg Fe^2+^/L	±1.0–±3.7 μg Fe^2+^/L	Yes	Battery	Yes	Yes	Yes
HACH Iron DR300 [50]	EUR 952	LED	Iron	-	0–5 mg Fe^2+^/L	±1.0 ± 0.2 mg/L Fe	-	Yes	Battery	Yes	Yes	Yes
HANNA HI-721 [51]	EUR 65	LED	Iron	-	0–5 mg Fe^2+^/L	±0.01 mg Fe^2+^/L	±0.04 mg Fe^2+^/L	Yes	Battery	Yes	No	No
Phytoplankton Fluorescence [52]	USD 150	LED	Phytoplankton	-	0.029 to 32.6 μg cla/L	-	-	Yes	Battery	No	No	No
Smart Turbidimeter [49]	EUR 8.30	LED	Turbidimetry	-	0–200 NTU	-	-	Yes	Battery	No	No	No
Water Turbidity Monitoring System [53]	-	LED	Turbidimetry	-	0–40 NTU	-	-	Yes	-	No	No	No
Photoelectrochemical detection of L-Dopa detector [54]	-	LED	L-Dopa	31.8 μA/L mmol	20 up to 190 μmol/L	-	-	Yes	-	Yes	No	No
FOS-SI-LOV [55]	-	LED	Free chlorine	-	10 to 400 μg/L	-	-	Yes	-	Yes	No	No

## 5. Conclusions and Future Perspectives

The prototype of a stand-alone portable iron measurement system (IMS) capable of determining concentrations of Fe^2+^ in freshwater has been presented. Measurement of absorbances necessary for computation of Fe^2+^ concentration in water samples was performed through processing attenuated spectroscopic data obtained from the TCS34725 RGBC photodiode, which senses the green channel component of radiation emitted from the RGB LED. An optoelectronic instrument with high accuracy and precision capable of repeatable measurements was developed based on measured intensity of integrated radiation in the range of 470–600 nm. Use of low-cost components enabled production of a cost-effective instrument compared to the commercially available ones, and its modular characteristics will facilitate further calibrations for the LED channels not explored in this paper (red and blue). The linearity of the IMS response function to different Fe^2+^ concentrations has been verified. Comparison of the IMS results against the Nicolet Evolution 300 spectrophotometer, considered as the reference instrument, revealed overestimation of absorbance values obtained with the prototype device that should be considered in the calibration function. The *t*-tests comparing the slopes of different measurement series show high linear agreement between the two instruments in the four series of measurements. The novel IMS instrument presented better measurement performance in terms of precision and accuracy than the Nicolet Evolution 300. Recovery analysis was successfully completed using natural water. The instrument successfully recovered iron concentrations from freshwater, showing its applicability in monitoring water bodies. The developed solid reagent presented very good performance on the prepared water solutions, making the portability of the equipment more effective. The next step in this process is to validate the solid reagent method for iron determination in the field. The IMS showed better performance than a similar state-of-the-art device, such as the one presented in 5145]; it is also portable and suitable for use in the field in near-real-time conditions. Further developments are ongoing to calibrate the instrument for use of the red and blue channels for determination of other water quality parameters, specifically lead concentration.

## Figures and Tables

**Table 1:** Nicolet Evolution 300 spectrophotometer characteristics.

Parameter	Value
Holographic grating	1200 lines/mm, blazed at 240 nm
Maximum resolution	0.5 nm
Range	190 to 1100 nm
Accuracy	±0.20 nm (546.11 nm Hg emission line) ±30 nm (190 to 900 nm)
Repeatable peak separation of repetitive scanning of Hg line source	<0.10 nm
Standard deviation of 10 measurements	<0.05 nm
Accuracy of instrument	1A: ±0.004 A 2A: ±0.004 A 3A: ±0.006 A
Repeatability of light intensity measurement	1A: ±0.0025 A
Drift	<0.0005 Abs/h at 500 nm, 2.0 nm SBW, 2 h warmup
Baseline flatness	±0.0015 A (200–800 nm), 2.0 nm SBW, smoothed

**Table 2:** Uncertainty analysis of standard solutions for IMS and Nicolet Evolution 300 in first and second series of measurements.

Standard Solution (µg Fe^2+^/L)	Standard Sample Uncertainty (µg Fe^2+^/L)	First Series						Second Series					
		IMS (µg Fe^2+^/L)	IMS Prec. (µg Fe^2+^/L)	IMS Uncert. (µg Fe^2+^/L)	Nicolet (µg Fe^2+^/L)	Nicolet Prec. (µg Fe^2+^/L)	Nicolet Uncert. (µg Fe^2+^/L)	IMS (µg Fe^2+^/L)	IMS Prec. (µg Fe^2+^/L)	IMS Uncert. (µg Fe^2+^/L)	Nicolet (µg Fe^2+^/L)	Nicolet Prec. (µg Fe^2+^/L)	Nicolet Uncert. (µg Fe^2+^/L)
25	±0.25	-		-	-		-	28.7	±88.3	±9.4	28.2	±795.2	±28.2
50	±0.50	54.8	±27.0	±5.2	54.7	±275.5	±16.6	44.4	±22.0	±4.7	41.3	±795.2	±28.2
75	±0.75	-	-	-	-	-	-	75.6	±36.0	±6.0	79.4	±795.2	±28.2
100	±1.00	98.4	±25.0	±5.0	98.1	±275.5	±16.6	97.0	±21.1	±4.6	98.6	±795.2	±28.2
125	±1.25	-	-	-	-	-	-	122.6	±49.0	±7.0	124.1	±795.2	±28.2
150	±1.50	-	-	-	-	-	-	152.2	±36.0	±6.0	149.7	±795.2	±28.2
200	±2.00	196.6	±18.4	±4.3	196,0	±275.5	±16.6	201.5	±28.0	±5.3	200.9	±795.2	±28.2
500	±5.00	513.8	±28.0	±5.3	511.2	±275.5	±16.6	-	-	-	-	-	-
1000	±10.00	993.3	±27.0	±5.2	994.9	±275.5	±16.6	-	-	-	-	-	-

**Table 3:** Uncertainty analysis of standard solutions for IMS and Nicolet Evolution 300 in third and fourth series of measurements.

Standard Solution (µg Fe^2+^/L)	Standard Sample Uncertainty (µg Fe^2+^/L)	Third Series						Fourth Series					
		IMS µg (Fe^2+^/L)	IMS Prec. (µg Fe^2+^/L)	IMS Uncert. (µg Fe^2+^/L)	Nicolet (µg Fe^2+^/L)	Nicolet Prec. (µg Fe^2+^/L)	Nicolet Uncert. (µg Fe^2+^/L)	IMS (µg Fe^2+^/L)	IMS Prec. (µg Fe^2+^/L)	IMS Uncert. (µg Fe^2+^/L)	Nicolet (µg Fe^2+^/L)	Nicolet Prec. (µg Fe^2+^/L)	Nicolet Uncert. (µg Fe^2+^/L)
25	±0.25	25.4	±13.6	±3.7	25.3	±470.8	±21.7	21.27	±46.2	±6.8	27.4	±750.6	±27.4
50	±0.50	50.3	±9.0	±3.0	48.6	±470.8	±21.7	48.58	±39.6	±6.3	43.3	±750.6	±27.4
75	±0.75	73.3	±4.0	±2.0	77.7	±470.8	±21.7	-	-	-	-	-	-
100	±1.00	101.0	±5.2	±2.3	95.2	±470.8	±21.7	101.19	±193.2	±13.9	91,0	±750.6	±27.4
125	±1.25	122.3	±3.6	±1.9	124.3	±470.8	±21.7	-	-	-	-	-	-
150	±1.50	154.0	±4.8	±2.2	159.3	±470.8	±21.7	158.1	±163.8	±12.8	159.9	±750.6	±27.4
200	±2.00	198.4	±1.0	±1.0	194.2	±470.8	±21.7	193.4	±46.2	±6.8	197.0	±750.6	±27.4

**Table 4:** Measurement of absorbance in first series of iron standard samples.

Sample Conc. (Fe^2+^/L)	IMS Mean Abs.	Nicolet Abs. (525 nm)	IMS Variance
50	0.010	0.011	2.06 × 10^−8^
100	0.016	0.019	2.59 × 10^−8^
200	0.032	0.037	4.62 × 10^−8^
500	0.081	0.095	1.96 × 10^−8^
1000	0.157	0.184	2.18 × 10^−8^

**Table 5:** Measurements of absorbance in second series of iron standard samples.

Sample Conc. (µg Fe^2+^/L)	IMS Mean Abs.	Nicolet Abs. (525 nm)	IMS Variance	Retrieved (µg Fe^2+^/L)	Recovery (%)
25	0.003	0.004	2.37 × 10^−7^	27.62	110.50
50	0.005	0.006	2.46 × 10^−8^	51.97	103.95
75	0.010	0.012	6.15 × 10^−8^	74.49	99.32
100	0.013	0.015	2.21 × 10^−8^	101.71	101.71
125	0.016	0.019	9.99 × 10^−8^	122.52	98.02
150	0.021	0.023	6.12 × 10^−8^	153.54	102.36
200	0.027	0.031	3.96 × 10^−8^	197.09	98.54

**Table 6:** Measurements of absorbance in third series of iron standard samples.

Sample Conc. (µg Fe^2+^/L)	IMS Mean Abs.	Nicolet Abs. (525 nm)	IMS Variance
25	0.004	0.007	5.47 × 10^−8^
50	0.008	0.011	8.30 × 10^−8^
75	0.012	0.016	1.32 × 10^−7^
100	0.016	0.019	1.17 × 10^−7^
125	0.019	0.024	1.35 × 10^−7^
150	0.024	0.030	1.19 × 10^−7^
200	0.031	0.036	3.58 × 10^−7^

**Table 7:** Measurements of absorbance in fourth series of iron standard samples.

Sample Conc. (µg Fe^2+^/L)	IMS Mean Abs.	Nicolet Abs. (525 nm)	IMS Variance
25	0.003	0.004	1.30 × 10^−7^
50	0.007	0.007	1.03 × 10^−7^
100	0.015	0.016	8.63 × 10^−7^
150	0.024	0.029	7.14 × 10^−7^
200	0.030	0.036	1.31 × 10^−7^

## Data Availability

Not applicable.

## References

[1] Yaqoob A., Parveen T., Umar K., Ibrahim M. (2020). Role of Nanomaterials in the Treatment of Wastewater: A Review. Water.

[2] Albert J., Destouni G., Duke-Sylvester S., Magurran A., Oberdorff T., Reis R., Winemiller K., Ripple W. (2021). Scientists’ warning to humanity on the freshwater biodiversity crisis. Ambio.

[3] (1988). Council Directive 98/83/EC of 3 November 1998 on the quality of water intended for human consumption. Off. J. Eur. Communities.

[4] Ji Y., Wu J., Wang Y., Elumalai V., Subramani T. (2020). Seasonal Variation of Drinking Water Quality and Human Health Risk Assessment in Hancheng City of Guanzhong Plain, China. Expo. Health.

[5] Khalid S., Shahid M., Natasha, Shah A., Saeed F., Ali M., Qaisrani S., Dumat C. (2020). Heavy metal contamination and exposure risk assessment via drinking groundwater in Vehari, Pakistan. Environ. Sci. Pollut. Res..

[6] Prasad S., Yadav K., Kumar S., Gupta N., Cabral-Pinto M., Rezania S., Radwan N., Alam J. (2021). Chromium contamination and effect on environmental health and its remediation: A sustainable approaches. J. Environ. Manag..

[7] Bashir I., Lone F., Bhat R., Mir S., Dar Z., Dar S. (2020). Concerns and threats of contamination on aquatic ecosystems. Bioremediation and Biotechnology.

[8] Adesakin T., Oyewale A., Bayero U., Mohammed A., Aduwo I., Ahmed P., Abubakar N., Barje I. (2020). Assessment of bacteriological quality and physico-chemical parameters of domestic water sources in Samaru community, Zaria, Northwest Nigeria. Heliyon.

[9] Yaqoob A., Ahmad H., Parveen T., Ahmad A., Oves M., Ismail I., Qari H., Umar K., Ibrahim M.M. (2020). Recent Advances in Metal Decorated Nanomaterials and Their Various Biological Applications: A Review. Front. Chem..

[10] Kotalik C., Cadmus P., Clements W. (2019). Indirect Effects of Iron Oxide on Stream Benthic Communities: Capturing Ecological Complexity with Controlled Mesocosm Experiments. Environ. Sci. Technol..

[11] Liu J., Fan Y., Yang Z., Wang Z., Guo C. (2018). Iron and Alzheimer’s Disease: From Pathogenesis to Therapeutic Implications. Front. Neurosci..

[12] Lane D., Ayton S., Bush A. (2018). Iron and Alzheimer’s Disease: An Update on Emerging Mechanisms. J. Alzheimers Dis..

[13] Kenkhuis B., Somarakis A., de Haan L., Dzyubachyk O., IJsselsteijn M., de Miranda N., Lelieveldt B., Dijkstra J., van Roon-Mom W., Höllt T. (2021). Iron loading is a prominent feature of activated microglia in Alzheimer’s disease patients. Acta Neuropathol. Commun..

[14] Sensi S., Granzotto A., Siotto M., Squitti R. (2018). Copper and Zinc Dysregulation in Alzheimer’s Disease. Trends Pharmacol. Sci..

[15] Ndayisaba A., Kaindlstorfer C., Wenning G. (2019). Iron in neurodegeneration—Cause or consequence?. Front. Neurosci..

[16] Li K., Reichmann H. (2016). Role of iron in neurodegenerative diseases. J. Neural Transm..

[17] Kempf T., Wollert K. (2020). Iron and atherosclerosis: Too much of a good thing can be bad. Eur. Heart J..

[18] Cornelissen A., Guo L., Sakamoto A., Virmani R., Finn A.V. (2019). New insights into the role of iron in inflammation and atherosclerosis. EBioMedicine.

[19] Liu J., Li Q., Yang Y., Ma L. (2020). Iron metabolism and type 2 diabetes mellitus: A meta-analysis and systematic review. J. Diabetes Investig..

[20] Sanjeevi N., Freeland-Graves J., Beretvas N., Sachdev P. (2018). Trace element status in type 2 diabetes: A meta-analysis. J. Clin. Diagn. Res..

[21] Zhou X., Zhang K., Cen C., Wu J., Wu X. (2021). Roles of metal ions in regulating the formation of a drinking water odorant (2,3,6-trichloroanisole) by Sphingomonas ursincola in drinking water. Sci. Total Environ..

[22] Liew C., Li X., Zhang H., Lee H. (2018). A fully automated analytical platform integrating water sampling-miniscale-liquid-liquid extraction-full evaporation dynamic headspace concentration-gas chromatography-mass spectrometry for the analysis of ultraviolet filters. Anal. Chim. Acta.

[23] Melo M., Mota F., Albuquerque V., Alexandria A. (2019). Development of a Robotic Airboat for Online Water Quality Monitoring in Lakes. Robot.

[24] Cassivi A., Tilley E., Waygood E., Dorea C. (2021). Evaluating self-reported measures and alternatives to monitor access to drinking water: A case study in Malawi. Sci. Total Environ..

[25] Didukh-Shadrina S., Losev V., Samoilo A., Trofimchuk A., Nesterenko P. (2019). Determination of metals in natural waters by inductively coupled plasma optical emission spectroscopy after preconcentration on silica sequentially coated with layers of polyhexamethylene guanidinium and sulphonated nitrosonaphthols. Int. J. Anal. Chem..

[26] Mesko M., Balbinot F., Scaglioni P., Nascimento M., Picoloto R., da Costa V. (2020). Determination of halogens and sulfur in honey: A green analytical method using a single analysis. Anal. Bioanal. Chem..

[27] Rocha D., Maringolo V., Araújo A., Amorim C., Montenegro M. (2021). An overview of Structured Biosensors for Metal Ions Determination. Chemosensors.

[28] Hopwood M., Birchill A., Gledhill M., Achterberg E., Klar J., Milne A. (2017). A Comparison between four analytical methods for the measurement of Fe(II) at nanomolar concentrations in coastal seawater. Front. Mar. Sci..

[29] DR300 Pocket Colorimeter, Iron, Ferrover, with Box. Hach USA. https://www.hach.com/dr300-pocket-colorimeter-iron-ferrover-with-box/product?id=55321383871.

[30] Model DC1500 Iron Colorimeter Lab. https://lamotte.com/model-dc1500-iron-colorimeter-lab.

[31] Kareem H., Dunaev D. The Working Principles of ESP32 and Analytical Comparison of using Low-Cost Microcontroller Modules in Embedded Systems Design. Proceedings of the 2021 4th International Conference on Circuits, Systems and Simulation (ICCSS).

[32] Maier A., Sharp A., Vagapov Y. Comparative analysis and practical implementation of the ESP32 microcontroller module for the internet of things. Proceedings of the 2017 Internet Technologies and Applications (ITA).

[33] Dasgupta P., Bellamy H., Liu H., Lopez J., Loree E., Morris K., Petersen K., Mir K. (1993). Light emitting diode based flow-through optical absorption detectors. Talanta.

[34] Wang C., Li Z., Pan Z., Li D. (2018). A High-Performance Optoelectronic Sensor Device for Nitrate Nitrogen in Recirculating Aquaculture Systems. Sensors.

[35] Fitzhenry C., Jowett L., Roche P., Harrington K., Moore B., Paull B., Murray E. (2021). Portable analyser using two-dimensional ion chromatography with ultra-violet light-emitting diode-based absorbance detection for nitrate monitoring within both saline and freshwaters. J. Chromatogr. A.

[36] Fay C., Nattestad A. (2021). Advances in Optical Based Turbidity Sensing Using LED Photometry (PEDD). Sensors.

[37] Duffy G., Maguire I., Heery B., Nwankire C., Ducrée J., Regan F. (2017). PhosphaSense: A fully integrated, portable lab-on-a-disc device for phosphate determination in water. Sens. Actuators B Chem..

[38] Pal A., Kulkarni M., Gupta H., Ponnalagu R., Dubey S., Goel S. (2021). Portable and Autonomous Device for Real-time Colorimetric Detection: Validation for Phosphorous and Nitrite Detection. Sens. Actuators A Phys..

[39] Seetasang S., Kaneta T. (2021). Portable two-color photometer based on paired light emitter detector diodes and its application to the determination of paraquat and diquat. Microchem. J..

[40] Sarıkaya M., Ulusoy H., Morgul U., Ulusoy S., Tartaglia A., Yılmaz E., Soylak M., Locatelli M., Kabir A. (2021). Sensitive determination of Fluoxetine and Citalopram antidepressants in urine and wastewater samples by liquid chromatography coupled with photodiode array detector. J. Chromatogr. A.

[41] Duffy G., Maguire I., Heery B., Gers P., Ducrée J., Regan F. (2018). ChromiSense: A colourimetric lab-on-a-disc sensor for chromium speciation in water. Talanta.

[42] Yan J., Ren J., Ren L., Yang Y., Yang S., Ren T. (2019). A novel structure design and fabrication method for low liquid consumption and high precision device of colorimeter in water quality detection. Sens. Actuators A Phys..

[43] Oliveira G.C., Machado C., Inácio D., da Silveira Petruci J., Silva S. (2022). RGB color sensor for colorimetric determinations: Evaluation and quantitative analysis of colored liquid samples. Talanta.

[44] De Berg K., Maeder M., Clifford S. (2016). A new approach to the equilibrium study of iron(III) thiocyanates which accounts for the kinetic instability of the complexes particularly observable under high thiocyanate concentrations. Inorg. Chim. Acta.

[45] Amendola C., Lacerenza M., Pirovano I., Contini D., Spinelli L., Cubeddu R., Torricelli A., Re R. (2021). Optical characterization of 3D printed PLA and ABS filaments for diffuse optics applications. PLoS ONE.

[46] Hossain M., Biswas P., Rani S., Eskender S., Islam M., Chakma A., Canning J. (2022). Low-Cost 3D Printer Drawn Optical Microfibers for Smartphone Colorimetric Detection. Biosensors.

[47] Rice E.W., Baird R.B., Eaton A.D., Clesceri L.S. (2012). Standard Methods for the Examination of Water and Wastewater.

[48] (2008). Evaluation of Measurement Data—Guide to the Expression of Uncertainty in Measurement—100:2008.

[49] Parra L., Rocher J., Escrivá J., Lloret J. (2018). Design and development of low cost smart turbidity sensor for water quality monitoring in fish farms. Aquac. Eng..

[50] DR300 Pocket Colorimeter, Chlorine, Free + Total, LR/HR, with Box. https://www.hach.com/p-dr300-colorimeters-parameter/LPV445.97.00110.

[51] HI-721 High Range Iron Handheld Colorimeter—Checker. https://www.hannainstruments.co.uk/photometers/808-pocket-checker-for-iron-testing.

[52] Leeuw T., Boss E., Wright D. (2013). In situ Measurements of Phytoplankton Fluorescence Using Low Cost Electronics. Sensors.

[53] Mulyana Y., Hakim D. (2018). Prototype of Water Turbidity Monitoring System. IOP Conf. Ser. Mater. Sci. Eng..

[54] Neto S.Y., De Cássia Silva Luz R., Damos F. (2016). Visible LED light photoelectrochemical sensor for detection of L-Dopa based on oxygen reduction on TiO2 sensitized with iron phthalocyanine. Electrochem. Commun..

[55] Xiong Y., Tan J., Fang S., Wang C., Wang Q., Wu J., Chen J., Duan M. (2017). A LED-based fiber-optic sensor integrated with lab-on-valve manifold for colorimetric determination of free chlorine in water. Talanta.

